# Deep‐targeted exon sequencing reveals renal polymorphisms associate with postexercise hypotension among African Americans

**DOI:** 10.14814/phy2.12992

**Published:** 2016-10-10

**Authors:** Linda S. Pescatello, Elizabeth D. Schifano, Garrett I. Ash, Gregory A. Panza, Lauren Lamberti, Ming‐Hui Chen, Ved Deshpande, Amanda Zaleski, Paulo Farinatti, Beth A. Taylor, Paul D. Thompson

**Affiliations:** ^1^ Department of KinesiologyUniversity of ConnecticutStorrsConnecticut; ^2^ Institute for Systems GenomicsUniversity of ConnecticutStorrsConnecticut; ^3^ Department of StatisticsUniversity of ConnecticutStorrsConnecticut; ^4^ School of NursingYale UniversityNew HavenConnecticut; ^5^ Department of Preventive CardiologyHartford HospitalHartfordConnecticut; ^6^ Department of Physical Activity SciencesRio de Janeiro State UniversityRio de JaneiroBrazil

**Keywords:** Blood pressure, exercise, hypertension, polymorphism

## Abstract

We found variants from the Angiotensinogen‐Converting Enzyme (*ACE*), Angiotensin Type 1 Receptor (*AGTR1*), Aldosterone Synthase (*CYP11B2*), and Adducin (*ADD1*) genes exhibited intensity‐dependent associations with the ambulatory blood pressure (BP) response following acute exercise, or postexercise hypotension (PEH). In a validation cohort, we sequenced exons from these genes for their associations with PEH. Obese (30.9 ± 3.6 kg m^−2^) adults (*n* = 23; 61% African Americans [AF], 39% Caucasian) 42.0 ± 9.8 years with hypertension (139.8 ± 10.4/84.6 ± 6.2 mmHg) completed three random experiments: bouts of vigorous and moderate intensity cycling and control. Subjects wore an ambulatory BP monitor for 19 h. We performed deep‐targeted exon sequencing using the Illumina TruSeq Custom Amplicon kit. Variant genotypes were coded as number of minor alleles (#MA) and selected for further statistical analysis based upon Bonferonni or Benjamini–Yekutieli multiple testing corrected p‐values under time adjusted linear models for 19 hourly BP measurements per subject. After vigorous intensity over 19 h among *ACE*,*AGTR1*,*CYP11B2*, and *ADD1* variants passing multiple testing thresholds, as the #MA increased, systolic (SBP) and/or diastolic BP *decreased* 12 mmHg (*P* = 4.5E‐05) to 30 mmHg (*P* = 6.4E‐04) among AF only. In contrast, after moderate intensity over 19 h among *ACE* and *CYP11B2* variants passing multiple testing thresholds, as the #MA increased, SBP *increased* 21 mmHg (*P* = 8.0E‐04) to 22 mmHg (*P* = 8.2E‐04) among AF only. In this replication study, *ACE*,*AGTR1*,*CYP11B2*, and *ADD1* variants exhibited associations with PEH after vigorous, but not moderate intensity exercise among AF only. Renal variants should be explored further with a multi‐level “omics” approach for associations with PEH among a large, ethnically diverse sample of adults with hypertension.

## Introduction

High blood pressure (BP) is the leading risk factor for global disease burden (GBD 2013 Risk Factors Collaborators et al. 2015). The inherited tendency towards hypertension predominately resides in the kidneys via the regulation of fluid and electrolyte balance (Arnett et al. 2007; Padmanabhan et al. 2012; Basson et al. 2014; Reiter et al. 2015). Pharmacogenetic studies have identified variants in the renin‐angiotensin system (RAS) and other renal functional variants to alter the BP response to antihypertensive drug treatment (Maitland‐van der Zee et al. 2005; Su et al. 2007; Bozkurt et al. 2008; Konoshita and Genomic Disease Outcome Consortium (G‐DOC) Study Investigators 2011; Turner et al. 2012; Do et al. 2016). Aerobic exercise training lowers BP 5–7 mmHg among those with high BP, reductions that rival those with many first‐line antihypertensive medications (ALLHAT Officers and Coordinators for the ALLHAT Collaborative Research Group. The Antihypertensive and Lipid‐Lowering Treatment to Prevent Heart Attack Trial 2002, Brown et al. 2013). Thus, regular exercise participation is universally recommended as first‐line antihypertensive lifestyle therapy (Pescatello et al. 2015a,b).

We and others have proposed that the BP reductions attributed to aerobic exercise training are largely the result of the BP response to acute aerobic exercise, or postexercise hypotension (PEH) (Fitzgerald 1981; Wilcox et al. 1982; Pescatello et al. 1991, 2004a; Haskell 1994; Halliwill 2001; Pescatello and Kulikowich 2001; Thompson et al. 2001; Collier et al. 2008). PEH is the immediate reduction in BP that occurs after an isolated exercise session and persists for up to 24 h after exercise (Kenney and Seals 1993; Pescatello et al. 2004a, 2015b). Postexercise hypotension is of similar magnitude to the BP reductions that result from exercise training (Meredith et al. 1990; Jennings et al. 1991; Pescatello and Kulikowich 2001; Murray et al. 2006; Moker et al. 2014), and strongly correlated with the BP response to exercise training (Liu et al. 2012; Hecksteden et al. 2013; Tibana et al. 2015). Due to their biological, functional, and clinical relevance, genetic variants that associate with hypertension, renal function, and the BP response to pharmacotherapy and/or exercise training are logical candidates to explore for their associations with PEH (Bouchard 2011; Ash et al. 2013a).

We completed a series of studies investigating the role of renal polymorphisms *reported* to be associated with hypertension (Arnett et al. 2007; Padmanabhan et al. 2012; Basson et al. 2014; Reiter et al. 2015) and the BP response to pharmacotherapy (Maitland‐van der Zee et al. 2005; Su et al. 2007; Bozkurt et al. 2008; Konoshita and Genomic Disease Outcome Consortium (G‐DOC) Study Investigators 2011; Turner et al. 2012; Do et al. 2016) and/or exercise training (Kohno et al. 1997; Krizanova et al. 1998; Hagberg et al. 1999; Rankinen et al. 2000; Jones et al. 2006, 2007) for their associations with PEH (Blanchard et al. 2006; Pescatello et al. 2007, 2008; Ash et al. 2013a). In these candidate gene association studies, we found that Angiotensin 1 Converting Enzyme (*ACE*) rs4646994, Angiotensin II Type 1 Receptor (*AGTR1*) rs5186, and Aldosterone Synthase (*CYP11B2*) rs1799998 from the RAS, and Adducin 1 (*ADD1*) rs4961, a renal structural variant, exhibited intensity‐dependent associations with PEH among 50 Caucasian men with pre‐ to Stage 1 hypertension. We recently conducted a meta‐analysis of candidate gene association studies examining the BP response to acute (i.e., PEH) and chronic (i.e., training) aerobic exercise and found that Angiotensiogen (*AGT*) rs699 emerged as the only promising variant to explore further (Bruneau et al. 2015). This finding substantiates a major concern of candidate gene association studies that examine the response of health‐related phenotypes to exercise; that is, most statistically significant findings fail to replicate due to a variety of factors that include the lack of standardized protocols, statistical adjustments for multiple comparisons, and adequately powered samples (Bouchard 2011; Bouchard et al. 2012; Ash et al. 2013a; Bruneau et al. 2015; Mattsson et al. 2016).

Utilizing advances in genomic technology that emerged since undertaking our discovery phase PEH candidate gene association studies, we performed deep‐targeted exon sequencing of *ACE*,* AGT*,* AGTR1*,* CYP11B2*, and *ADD1* to determine if they harbored polymorphisms associated with PEH in a validation cohort.

## Materials and Methods

### Overview

**Figure 1:**
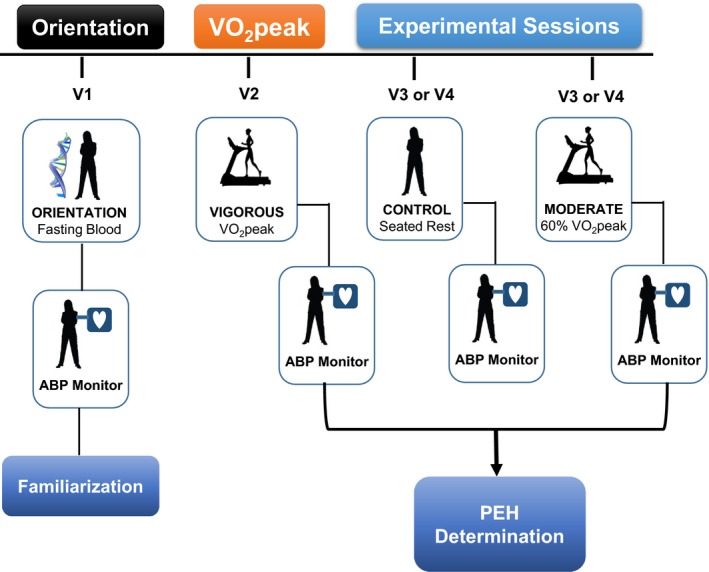
Study design overview. ABP, ambulatory blood pressure worn until the next morning; VO_2peak_, peak oxygen consumption as determined on the peak cardiopulmonary graded exercise stress test.

We employed the identical study design used in our discovery phase PEH studies that is overviewed in Figure 1 (Pescatello et al. 1991, 2004b, 2007, 2008; Blanchard et al. 2006; Eicher et al. 2010). At an orientation session, subjects (*n* = 23) provided a blood sample for deep‐targeted exon sequencing and a fasting cardiometabolic health profile. They left the laboratory wearing an ambulatory BP monitor until the next morning to acquaint them with wearing the technology (Ash et al. 2013b). Subjects then completed three randomly assigned acute experiments: a cardiopulmonary graded exercise test (GEST) on a cycle ergometer to obtain peak oxygen consumption (VO_2peak_) (VIGOROUS); 30 min of cycling at 60% VO_2peak_ (MODERATE); and a 30 min control session of seated rest (CONTROL). We measured BP for 20 min before and 45 min after the acute experiments. Subjects then left the laboratory wearing an ambulatory BP monitor over 19 h until the next morning.

### Subjects

Sedentary, defined as exercising ≤2 days per week, who were 18–55 years with pre‐ to stage 1 hypertension and a body mass index (BMI) ≥25 to <40 kg·m^−2^ were enrolled. Any medications that could potentially influence BP including inhaled or oral steroids, nonsteroidal anti‐inflammatory agents, aspirin, antihypertensive and hyperlipidemic medications, nutritional supplements besides one‐a‐day vitamin, cold medications, hormone‐altering contraception, or herbal supplements were stopped at least 4 weeks prior to testing. Subjects with osteoarthritis and orthopedic problems were not recruited if these conditions compromised their ability to complete the acute exercise experiments. Two participants with mild hypertension discontinued their antihypertensive medications with physician permission ≥6 weeks prior to study participation. Women were premenopausal and regularly menstruating. Subjects remained weight stable throughout study participation, defined as gaining or losing <2.25 kg of orientation body weight. Participants completed an informed consent approved by the Institutional Review Boards of the two participating sites.

### Body composition

Body mass index (kg·m^−2^) was calculated from body weight and height using a calibrated balance beam scale. Waist circumference was measured at the narrowest part of the torso using a nondistensible Guilick tape measure (Pescatello et al. 2013).

### Blood pressure

Blood pressure (BP) was measured using an automated BPTRU monitor (BPTRU Medical Devices; Coquitlam, Canada) according to American Heart Association standards (Pickering et al. 2005) at the orientation session to determine BP status. BP was also measured before the acute experiments every 2 min for 20 min in the nondominant arm with the automated BPTRU monitor and averaged as baseline BP. After the orientation session and the three experiments (i.e., CONTROL, MODERATE, VIGOROUS) using our standardized protocols (Pescatello et al. 1991, 2004b, 2007, 2008; Blanchard et al. 2006; Eicher et al. 2010), subjects were attached to the same Oscar2 ambulatory BP monitor (Oscar2 automatic noninvasive ambulatory BP monitor, Suntech Medical Instruments Inc., Raleigh, NC) calibrated to a mercury sphygmomanometer.

Subjects proceeded with normal daily activities and no formal exercise while the monitor recorded three ambulatory BP assessments per waking hour and two per sleep over 19 h. Subjects carried a journal, recording activities performed during each measurement, any unusual physical or emotional events, and their awake and sleep hours. We omitted ambulatory BP readings of systolic BP (SBP) >220 or <80 mmHg, or diastolic BP (DBP) >130 or <40 mmHg according to the manufacturer's exclusion criteria. Computerized ambulatory BP reports were acceptable if at least 80% of the potential BP readings were obtained. Ambulatory arterial stiffness index was calculated after CONTROL as 1 − (slope of DBP vs. SBP over 19 h) (Dolan et al. 2006).

### Acute experiments

Participants completed three randomly assigned acute experiments: a nonexercise control session of seated rest (CONTROL) and two cycle exercise bouts on an upright cycle ergometer (Monarch 839E Digital Cycle Ergometer, Stockholm, Sweden) at 60% VO_2peak_ (MODERATE) and 100% VO_2peak_ (VIGOROUS) (Fig. 1). All experiments were conducted at the same time of day to account for diurnal variation in BP, separated by a minimum of 48 h to avoid acute exercise effects, and completed within 1 month of beginning study participation. The same investigator measured heart rate (HR), SBP, and DBP for each subject and experiment. Subjects sat quietly for a 20 min baseline period at the start of each experimental session. During the baseline period, HR was recorded using a HR monitor (Polar Electro Inc., Port Washington, NY) every 2 min, whereas SBP and DBP were measured every other minute by auscultation. Each experiment was followed by a 45 min recovery period in the seated position with BP and HR measured every 2 min. Subjects were attached to the ambulatory BP monitor after the experiments until waking the next morning.

VIGOROUS (100% VO_2peak_) consisted of a peak cardiopulmonary GEST. VO_2peak_ was determined by breath‐by‐breath analysis of expired gases (ParvoMedicsTrueOne^®^ 2400 Metabolic Measurement System, ParvoMedics Inc., Sandy, UT). The GEST began with a resistance of 0.5 kp (30 W) and consisted of continuous cycling at a constant cadence (60 rev·min^−1^) with the resistance increased 0.5 kp every 2 min until volitional exhaustion. During the GEST, HR was recorded continuously with a 12‐lead electrocardiograph system (Marquette Case 8000, Jupiter, FL), and BP was measured every 2 min by auscultation. Results of the peak cardiopulmonary GEST (VIGOROUS) were used to calculate the workload of the other acute exercise experiment (MODERATE). Each volunteer performed the two remaining experiments in random order: nonexercise control and MODERATE (60% VO_2peak_). CONTROL was a 30 min session of seated rest. MODERATE consisted of a 5 min warm‐up of free‐wheeling with no resistance, 20 min of cycling at 60% VO_2peak_, and a 5 min cool down period to total 30 min. HR, SBP, and DBP were measured every 5 min during nonexercise control and MODERATE.

### Blood sampling and analysis

During the orientation session, fasting blood samples were drawn without stasis from an antecubital vein and centrifuged at 3400 × *g* at 23°C for 10–15 min. Serum and plasma samples were drawn in red top and EDTA vacutainer tubes, respectively. Serum and plasma samples were aliquoted into separate 1.8 mL nonpyrogenic storage tubes and frozen at −80°C until analysis. Glucose and insulin were determined by enzymatic/spectrophotometric methods. Homeostatsis model assessment, an indicator of insulin resistance, was then calculated (Matthews et al. 1985). Total cholesterol, triglycerides, and high‐density lipoprotein cholesterol were determined by enzymatic/spectrophometric assays, and low‐density lipoprotein cholesterol calculated with the Friedewald equation (Friedewald et al. 1972). Nitrite (NO_2_^−^)/Nitrate (NO_3_^−^), high sensitivity C‐reactive protein (CRP), endothelin 1–21, and plasma renin activity (PRA) were detected by enzymatic/spectrophotometric methods. Analyses were performed with two levels of quality control material. A blood sample for DNA was drawn in an EDTA purple top vacutainer tube that was centrifuged for white cell isolation and frozen at −80°C until DNA extraction.

### Targeted sequencing and variant calling

We then performed deep‐targeted exon sequencing of a prioritized panel of 41 genes that harbored polymorphisms *reported* to be associated with hypertension, the BP response to pharmacotherapy, and/or the BP response to PEH and exercise training (Ash et al. 2013a; Bruneau et al. 2015) using the Illumina TruSeq Custom Amplicon kit (Catalog# FC‐130‐1001, Illumina, San Diego, CA) (see Supplement Material for the prioritized panel of genes). The Illumina DesignStudio software was used to create probes for the generation of 1214 amplicons with a size range 225–275 bp. The TruSeq Custom Amplicon manifest file associated with this panel included Target ID, region, chromosome, and start and end hg19 reference coordinate positions. Sequencing libraries were prepared following the TruSeq Custom Amplicon Library Preparation Guide. DNA input mass for all libraries was 250 ng of DNA. Libraries were generated with dual indices (23 PCR cycles), followed by normalization and pooling. The library amplicon pool was sequenced using Illumina MiSeq version 2 reagents (250 paired‐end reads). 7.1 million pair‐end reads (6.8 million passing quality filter) were produced from the library amplicon pool. MiSeq Reporter Software (version 2.3.32), using the TruSeq Amplicon workflow was used to generate Fastq files and align reads to the hg19 human reference sequence using the Smith‐Waterman algorithm. Genome Analysis Toolkit (GATK) was use for variant calling (single‐nucleotide polymorphisms and small insertion/deletions) and the generation of variant calling files (VCF). For all further downstream analysis, a merged VCF was generated using VCFtools v 0.1.12b (Danecek et al. 2011) and custom R scripts (R v3.2.0). Only variants with FILTER = PASS were retained. For each defined amplicon target region, we calculated the total number of variants present per subject and each polymorphism's major and minor allele frequency for each subject.

### Statistical analysis

Descriptive statistics (Mean ± SD) were generated on study variables for the total sample and by ethnic group. Independent *t*‐tests determined if there were differences in subject descriptive characteristics between ethnic groups. Repeated measures analysis of covariance compared the BP response, defined as the change from baseline following exercise – change from baseline following control, at hourly intervals under ambulatory conditions with age and BMI as covariates and gender and ethnicity as fixed factors over 19 h. These statistical analyses were performed with SPSS 14.0 (Chicago, IL).

#### Renal variant screening

Variant genotypic values were coded as the number of minor alleles (#MA). Genotypic values for 645 variants from the 41 genes were analyzed (see Supplemental Material for the prioritized gene panel and variants that were exon sequenced). For each polymorphism, we fit a linear model for each race/ethnicity separately that included polynomial time (order 3), polymorphism under an additive model, and polymorphism x time interactions as covariates; the dependent variable, BP response, was defined as the change from baseline following exercise – change from baseline following control. Since there are 19 observations per subject (i.e., *n* = 9 Caucasians × 19 h = 171 observations; *n* = 14 African Americans [AF] × 19 h = 266 observations), we assumed a first‐order autoregressive (AR1) correlation structure. Residual errors within each subject are therefore correlated, but were independent across subjects. Bonferroni and Benjamini–Yekutieli (BY) (Benjamini and Yekutieli 2005) adjusted p‐values were calculated for each polymorphism, correcting for the total number of unique polymorphism profiles among the corresponding racial/ethnic group resulting in 300 polymorphisms for AF and 146 for Caucasians. Polymorphisms with Bonferroni adjusted *P*‐values <0.05 and/or BY adjusted *P*‐values <0.20 were identified; renal polymorphisms from our discovery phase PEH candidate gene association studies achieving these multiple testing thresholds were then considered for further statistical modeling and analysis. Renal genotype‐BP differences by the #MA after VIGOROUS and MODERATE compared to CONTROL are reported as the average change over 19 h with the associated p value resulting from the screening model that accounted for repeated measures over time.

#### Final multivariable regression models

For each renal polymorphism passing the multiple testing threshold, we selected model effects (i.e., covariates, order of time polynomial, additive versus dominant/recessive genetic models) and within‐subject correlation structure based on models fit with maximum likelihood (ML) using Akaike Information Criteria and likelihood ratio tests (LRT). Covariates that were marginally associated (*P* < 0.05) with the BP response were eligible to be included in the final models. Possible within subject correlation structures included compound symmetry, AR1, and independent structures, however, AR1 provided the best fit in all cases. On the basis of ML estimation using AR1 within‐subject correlation structure, we report the LRT p‐values and pseudopartial *R*‐squared measures (i.e., the partial proportion of variation explained [PVE]) for the polymorphism effects. We defined the PVE for a given model using the pseudo‐*R*‐squared measure *R* ^2^_m_ = 1 − (*L*_R_/*L*_U_)^2/*n*^, where *L*_R_ is the restricted maximized likelihood from a model containing only an intercept, *L*_U_ is the unrestricted maximized likelihood for the given model, and *n* is the number of subjects (Maddala 1983; Magee 1990). The partial PVE for each polymorphism was the difference between *R* ^2^_m_ for the final model (including the covariate and polymorphism effects) and *R* ^2^_m_ for a model with polymorphism effect(s) excluded (Schemper 1993). We also report the parameter estimates for the final models, obtained using the restricted maximum likelihood (REML) method. Statistical analysis was performed in R (screening) and SAS version 9.4 (final models).

### Combined annotation‐dependent depletion

For each polymorphism that passed the multiple testing threshold, we determined the combined annotation‐dependent depletion (CADD) score from www.cadd.gs.washington.edu (Kircher et al. 2014). CADD scores quantitatively prioritize functional, deleterious, and disease causal variants across a wide range of functional categories, effect sizes, and genetic architectures. The higher the CADD score, the more severe is the allelic substitution in terms of its causal variation and regulatory effects. CADD scores of ≥10 indicate that substitutions in a given polymorphism are predicted to be the 10% most deleterious substitutions in the human genome.

## Results

### Subjects

Subjects (*n* = 23) were middle‐aged, obese Caucasian men (39%) and AF (61%) men (*n* = 10) and women (*n* = 4) with hypertension (Table 1). Only 22.2% of the Caucasians reported a family history of hypertension, whereas 64.3% of the AF did (*P* = 0.049). The overall cardiometabolic health profile of the AF was more favorable than the Caucasians; however, only waist circumference (*P* = 0.015), total cholesterol (*P* = 0.027), and triglycerides (*P* = 0.003) achieved statistical significance between ethnic groups. Reference ranges indicated that on average NO_2_^−^/NO_3_^−^ (Ghasemi et al. 2010) were low among AF and normal‐high among Caucasians; CRP was high among AF and Caucasians (http://lsplinks.net/QUESTCRP); and endothelin (http://lsplinks.net/ALPCOBig) and PRA (Brossaud and Corcuff 2009) were normal among AF and Caucasians.

**Table 1:** Subject characteristics (X ± SD)

Variable	Caucasians (*n* = 9)	African Americans (*n* = 14)
Age (year)	45.1 ± 7.8	39.9 ± 10.6
Body mass index (kg·m^−2^)	30.5 ± 1.8	31.1 ± 4.5
Waist circumference (cm)	98.0 ± 7.2	88.3 ± 9.4*
Relative peak oxygen consumption (mL·kg^−1^·min^−1^)	29.7 ± 6.4	25.3 ± 5.7
Awake systolic blood pressure (mmHg)	139.3 ± 7.0	140.2 ± 12.3
Awake diastolic blood pressure (mmHg)	85.0 ± 5.1	84.3 ± 6.9
Glucose (mg·dL^−1^)	96.4 ± 12.2	97.2 ± 10.3
Ambulatory arterial stiffness index	0.415 + 0.930	0.391 + 0.143
Insulin (μIU·mL^−1^)	13.1 ± 10.1	9.4 ± 6.0
Homeostatic model of assessment	3.1 ± 2.2	2.3 ± 1.5
Total cholesterol (mg·dL^−1^)	207.8 ± 31.3	178.5 ± 27.3*
Low‐density lipoproteins (mg·dL^−1^)	129.4 ± 20.3	108.3 ± 32.7
High‐density lipoproteins (mg·dL^−1^)	44.1 ± 10.9	53.9 ± 14.8
Triglycerides (mg·dL^−1^)	170.8 ± 88.8	83.7 ± 35.8**
Nitrite (NO_2_^−^)/Nitrate (NO_3_^−^) (μmol·L^−1^)	23.3 ± 37.0	10.9 ± 13.1
C‐reactive protein (mg·dL^−1^)	1.1 ± 1.0	2.8 ± 3.5
Endothelin (pmol·L^−1^)	0.222 ± 0.213	0.378 ± 0.663
Plasma renin activity (ng·mL^−1^·h^−1^)	1.7 ± 1.1 (*n* = 2)	0.946 ± 0.840 (*n* = 8)

*P < 0.05; **P < 0.01.

### Blood pressure response

#### Overall

**Figure 2:**
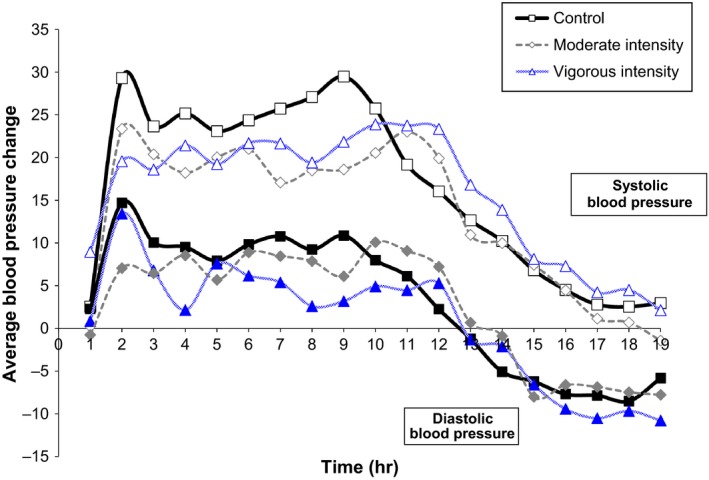
The average change in ambulatory systolic and diastolic blood pressure from baseline after aerobic exercise versus control at hourly intervals over 19 h. CONTROL, nonexercise session of seated rest; MODERATE, 60% VO_2peak_; VIGOROUS, VO_2peak_; *P* > 0.05 exercise versus nonexercise control.

Among the total sample, the SBP and DBP responses over 19 h were not different after VIGOROUS (SBP/DBP, −0.7 ± 13.4/−0.6 ± 7.7 mmHg) or MODERATE (−3.1 ± 8.0/−2.5 ± 5.7 mmHg) compared to control (*P* > 0.05) (Fig. 2). Furthermore, the SBP and DBP responses over 19 h were not different between Caucasians versus AF after VIGOROUS (SBP/DBP, 0.6 ± 10.1/−0.2 ± 5.3 mmHg versus −1.5 ± 15.5/−0.9 ± 9.1 mmHg) or MODERATE (−3.0 ± 6.6/−1.6 ± 5.2 mmHg versus −3.2 ± 9.0/−3.0 ± 6.1 mmHg), respectively, (*P* > 0.05).

#### By number of renal variant minor alleles

When the SBP and DBP responses over 19 h after VIGOROUS and MODERATE compared to control were examined by renal variant #MA, a very different scenario emerged regarding the BP response between the ethnic groups versus the overall BP response. After VIGOROUS, as the #MA increased from 0 to 1 or 2 depending on the variant, systolic (SBP) and/or diastolic (DBP) BP decreased after exercise versus control over 19 h (Table 2). For, *ACE* rs1055086, SBP decreased by −24.5 mmHg (*P* = 3.98E‐06) and DBP decreased by −16.3 mmHg (*P* = 1.04E‐07); *AGTR1* rs74662294, SBP decreased by −30.4 mmHg (*P* = 6.4E‐04) and DBP decreased by −20.3 mmHg (*P* = 9.73E‐05); *CYP11B2* rs4546, DBP decreased by −22.5 mmHg (*P* = 4.91E‐05); *CYP11B2* rs4537, SBP decreased by −30.4 mmHg (*P* = 6.4E‐04) and DBP decreased by −20.3 mmHg (9.73E‐05); *ADD1* rs16843169, SBP decreased by −20.4 mmHg (*P* = 2.0E‐03); and *ADD1* rs6833874, SBP decreased by −19.5 mmHg (*P* = 1.96E‐04) and DBP decreased by −12.2 mmHg (*P* = 4.5E‐05) among AF but not Caucasians. In contrast, after MODERATE, as the #MA increased from 0 to 1 or 2 depending on the variant, SBP increased after exercise versus control over 19 h (Table 3). For, *ACE* rs3730036, SBP increased by 20.8 mmHg (*P* = 8.0E‐04); *CYP11B2* rs6432, SBP increased by 20.8 mmHg (*P* = 8.0E‐04); and *CYP11B2* rs3802228, SBP increased by 21.6 mmHg (*P* = 8.2E‐04) among AF but not Caucasians.

**Table 2:** The blood pressure response (X ± SD)^1^ after versus before VIGOROUS exercise compared to control over 19 h by # of Minor Alleles and Racial/Ethnic Group

Gene name (Gene symbol)	Chromosome (Chr) location	Variant	Function	CADD score	African Americans (*n* = 14)			Caucasians (*n* = 9)		
					# Minor Alleles	SBP	DBP	# Minor Alleles	SBP	DBP
Angiotensinogen Converting Enzyme (*ACE*)	Chr17q23.3	rs1055086	3′ UTR	NA	0 (*n* = 11) 1 (n = 3) 2 (n = 0)	**3.7 ± 10.1** −**20.8 ± 18.2** NA	**2.6 ± 5.5** −**13.7 ± 8.4** NA	0 (*n* = 4) 1 (*n* = 3) 2 (*n* = 2)	2.0 ± 6.5 −1.6 ± 15.3 1.0 ± 14.1	−0.3 ± 3.8 0.4 ± 7.0 −0.8 ± 9.3
Angiotensin Type 1 Receptor (*AGTR1*)	Chr3q24	rs74662294	Intronic/Intergenic/Upstream	9.038	0 (*n* = 13) 1 (*n* = 1) 2 (*n* = 0)	**0.6 ± 13.7** −**30.0 ± NA** NA	**0.6 ± 7.6** −**19.7 ± NA** NA	0 (*n* = 9) 1 (*n* = 0) 2 (*n* = 0)	0.6 ± 10.1 NA NA	−0.2 ± 5.4 NA NA
Aldosterone Synthase (*CYP11B2*)	Chr8p24.3	rs4546*	Synonymous/Coding Transcript	1.736	0 (*n* = 8) 1 (*n* = 5) 2 (*n* = 1)	2.3 ± 10.5 −1.9 ± 18.9 −29.8 ± NA	**2.8 ± 5.2** −**3.0 ± 10.0** −**19.7 ± NA**	0 (*n* = 4) 1 (*n* = 3) 2 (*n* = 2)	−2.3 ± 5.6 −2.6 ± 14.8 11.1 ± 0.2	1.6 ± 6.5 −3.0 ± 2.1 0.5 ± 7.5
		rs72552275	Synonymous/Coding Transcript	1.736						
		rs4539	Missense/Coding Transcript	0.001						
		rs4537	Missense/Coding Transcript	0.001	0 (*n* = 13) 1 (*n* = 1) 2 (*n* = 0)	**0.6 ± 13.7** −**29.8 ± NA** NA	**0.6 ± 7.8** −**19.7 ± NA** NA	0 (*n* = 9) 1 (*n* = 0) 2 (*n* = 0)	0.6 ± 10.1 NA NA	−0.2 ± 5.4 NA NA
Adducin (*ADD1*)	Chr4p16.3	rs16843169*	Synonymous/Coding Transcript	13.93	0 (*n* = 12) 1 (*n* = 2) 2 (*n* = 0)	**1.4 ± 14.0** −**19.0 ± 15.2** NA	0.1 ± 7.7 −6.6 ± 18.5 NA	0 (*n* = 9) 1 (*n* = 0) 2 (*n* = 0)	0.6 ± 10.1 NA NA	−0.2 ± 5.4 NA NA
		rs4969	3′ UTR/Intergenic/Downstream	4.696						
		rs36038921	3′ UTR/Intergenic	0.112						
		rs4741	3′ UTR/Intergenic/Downstream	0.298						
		rs6833874*	Intronic/Transcript	2.195	0 (*n* = 10)	**4.1 ± 10.6**	**2.6 ± 6.0**	0 (*n* = 9)	0.6 ± 10.1	−0.2 ± 5.4
		rs4966	3′ UTR/Intergenic/Downstream	1.77	1 (*n* = 4)	−**15.5 ± 18.4**	−**9.8 ± 10.4**	1 (*n* = 0)	NA	NA
					2 (*n* = 0)	NA	NA	2 (*n* = 0)	NA	NA

Chromosome location and function obtained from SNAP, SNP Annotation and Proxy Search www.broadinstitute.org/mpg/snap/ldsearch.php; CADD, Combined Annotation‐Dependent Depletion, a score that prioritizes causal variation and regulatory effects www.cadd.gs.washington.edu; SBP, Systolic Blood Pressure; DBP, Diastolic Blood Pressure; UTR, Untranslated Region; NA, Not Available; *Multiple variants with the same # minor allele values.

^1^X and SD are computed as the average and standard deviation, respectively, of the subject‐level BP response averaged over 19 h. Bolded values correspond to significant effects after multiple testing adjustment.

**Table 3:** The blood pressure response (X ± SD)^1^ after versus before MODERATE compared to control over 19 h by # of Minor Alleles and Racial/Ethnic Group

Gene name (Gene symbol)	Chromosome (Chr) location	Variant	Function	CADD score	African Americans (*n* = 14)			Caucasians (*n* = 9)		
					# Minor Alleles	SBP	DBP	# Minor Alleles	SBP	DBP
Angiotensinogen Converting Enzyme (*ACE*)	Chr17q23.3	rs3730036*	Synonymous/Missense/Coding Transcript	17.2	0 (*n* = 13) 1 (*n* = 1) 2 (*n* = 0)	−**4.7 ± 7.3** **16.1 ± NA** NA	−3.5 ± 6.0 3.9 ± NA NA	0 (*n* = 9) 1 (*n* = 0) 2 (*n* = 0)	−3.0 ± 6.6 NA NA	−1.6 ± 5.2 NA NA
		rs3730038	Intronic/Transcript	1.614						
		rs3730042	Intronic/Transcript	1.834						
		rs28730840	Intronic/Transcript	3.486						
Aldosterone Synthase (*CYP11B2*)	Chr8p24.3	rs6432*	Intronic/Regulatory	0.38	0 (*n* = 13) 1 (*n* = 1) 2 (*n* = 0)	−**4.7 ± 7.3** **16.1 ± NA** NA	−3.5 ± 6.0 3.9 ± NA NA	0 (*n* = 8) 1 (*n* = 1) 2 (*n* = 0)	−2.6 ± 6.9 −6.6 ± NA NA	−2.1 ± 5.4 1.9 ± NA NA
		rs72499120	3′ UTR/Intronic/Transcript	0.225						
		rs4545	Missense/Coding Transcript	0.22						
		rs4536	Synonymous	NA						
		rs3802228*	3′ UTR	NA	0 (*n* = 11)	−**5.5 ± 7.3**	−3.9 ± 6.4	0 (*n* = 2)	1.6 ± 11.4	−1.7 ± 11.6
		rs7463212	Downstream	NA	1 (*n* = 2)	−**0.5 ± 8.3**	−1.5 ± 2.7	1 (*n* = 4)	−4.5 ± 7.1	−2.9 ± 4.5
					2 (*n* = 1)	**16.8 ± NA**	3.9 ± NA	2 (*n* = 3)	−4.1 ± 2.3	0.1 ± 1.6

Chromosome location and function obtained from SNAP, SNP Annotation and Proxy Search www.broadinstitute.org/mpg/snap/ldsearch.php; CADD, Combined Annotation‐Dependent Depletion, a score that prioritizes causal variation and regulatory effects www.cadd.gs.washington.edu; SBP, Systolic Blood Pressure; DBP, Diastolic Blood Pressure; UTR, Untranslated Region; NA, Not Available; *Multiple variants with the same # minor allele values.

^1^X and SD are computed as the average and standard deviation, respectively, of the subject‐level BP response averaged over 19 h. Bolded values correspond to significant effects after multiple testing adjustment.

### Proportion of variance explained

Table 4 contains the PVE for the SBP and DBP response to VIGOROUS and MODERATE among AF for the final multivariable regression models without the renal variants, and the partial PVE for each renal polymorphism after accounting for the other covariates in the model. For the SBP response to VIGOROUS, resting ambulatory SBP over 19 h, age, resting ambulatory arterial stiffness index over 19 h, and time (order 3) accounted for 92.5% of the variation. When the other covariates in the model were accounted for, the individual renal variants explained an additional 2.6% (*P* = 0.0143) to 5.8% (*P* < 0.0001) of the variation. For the DBP response to VIGOROUS, fasting triglycerides, gender, and endothelin accounted for 85.8% of the variation. When the other covariates in the model were accounted for, the individual renal variants explained an additional 3.6% (*P* = 0.0429) to 7.6% (*P* = 0.0011) of the variation. For the SBP response to MODERATE, insulin accounted for 66.2% of variation. When insulin was accounted for, the individual renal variants explained an additional 6.9% (*P* = 0.0736) to 8.6% (*P* = 0.0429) of the variation.

**Table 4:** The proportion of variance explained in the multivariable regression models for the systolic and diastolic blood pressure response following VIGOROUS and MODERATE among African Americans

VIGOROUS				
SBP				
Gene	Polymorphism	Model^1^	PVE^2^	*P* value^3^
None	None	BP response = −1.7872 + 2.1590*time + 0.0207*time^2 − 0.0320*time^3 + 16.4343*log(AASICONT) + 0.6782*Orientation 19 h SBP + 0.5765*Age	0.9252	–
Gene	Polymorphism	Model^1^	Partial PVE^2^	*P* value^3^
*ACE*	rs1055086	BP response = 2.5653 + 2.1353*time + 0.0076*time^2 − 0.0315*time^3 + 11.2080*log(AASICONT) + 0.6832*Orientation 19 h SBP + 0.4349*Age − 18.8394*SNP	0.0581	<0.0001
*AGTR1*	rs74662294	BP response = −0.2841 + 1.5349*time + 0.0091*time^2 − 0.0210*time^3 + 11.1560*log(AASICONT) + 0.6298*Orientation 19 h SBP + 0.5368*Age − 18.5855*SNP + 8.5552*SNP*time + .0618*SNP*time^2 − 0.1505*SNP*time^3	0.0562	0.0006
*CYP11B2*	rs4546	BP response = 2.3621 + 0.8792*time + 0.0024*time^2 − 0.0104*time^3 + 12.8728*log(AASICONT) + 0.6632*Orientation 19 h SBP + 0.5562*Age − 8.0779*SNP + 2.5435*SNP*time + 0.0276*SNP*time^2 − 0.0427*SNP*time^3	0.0457	0.0103
*CYP11B2*	rs4546^4^	BP response = −0.2841 + 1.5349*time + 0.0091*time^2 − 0.0210*time^3 + 11.1560*log(AASICONT) + 0.6298*Orientation 19 h SBP + 0.5368*Age − 18.5855*SNPr + 8.5552*SNPr*time + 0.0618*SNPr*time^2 − 0.1505*SNPr*time^3	0.0562	0.0006
*CYP11B2*	rs4537	BP response = −0.2841 + 1.5349*time + 0.0091*time^2 − 0.0210*time^3 + 11.1560*log(AASICONT) + 0.6298*Orientation 19 h SBP + 0.5368*Age − 18.5855*SNP + 8.5552*SNP*time + 0.0618*SNP*time^2 − 0.1505*SNP*time^3	0.0562	0.0006
*ADD1*	rs16843169	BP response = 0.3502 + 1.2629*time − 0.0114*time^2 − 0.0164*time^3 + 13.6612*log(AASICONT) + 0.5605*Orientation 19 h SBP + 0.6071*Age − 14.0158*SNP + 6.2030*SNP*time + 0.1861*SNP*time^2 − 0.1079*SNP*time^3	0.0520	0.0023
*ADD1*	rs6833874	BP response = 1.2998 + 2.1528*time + 0.0172*time^2 − 0.0318time^3 + 12.5206*log(AASICONT) + 0.5062*Orientation 19 h SBP + 0.5701*Age − 10.5065*SNP	0.0261	0.0143
DBP				
Gene	Polymorphism	Model^1^	PVE^2^	*P* value^3^
None	None	BP response = −3.1187 − 4.3889*log(Endothelin) − 8.5055*log(TRIG) + 7.6938*Gender	0.8577	–
Gene	Polymorphism	Model^1^	Partial PVE^2^	*P* value^3^
*ACE*	rs1055086	BP response = −0.6785 − 2.6365*log(Endothelin) − 8.8759*log(TRIG) + 2.6364*Gender − 10.9469*SNP	0.0608	0.0052
*AGTR1*	rs74662294	BP response = −1.7776 − 4.0777*log(Endothelin) − 5.5915*log(TRIG) + 6.8446*Gender − 15.2493*SNP	0.0760	0.0011
*CYP11B2*	rs4546	BP response = 0.5261 − 3.9426*log(Endothelin) − 6.4122*log(TRIG) + 5.8313*Gender − 6.2073*SNP	0.0741	0.0013
*CYP11B2*	rs4546^4^	BP response = −1.7776 − 4.0777*log(Endothelin) − 5.5915*log(TRIG) + 6.8446*Gender − 5.2493*SNPr	0.0760	0.0011
*CYP11B2*	rs4537	BP response = −1.7776 − 4.0777*log(Endothelin) − 5.5915*log(TRIG) + 6.8446*Gender − 15.2493*SNP	0.0760	0.011
*ADD1*	rs16843169	BP response = −1.7941 − 4.6642*log(Endothelin) − 8.2145*log(TRIG) + 6.5921*Gender − 7.0455*SNP	0.0361	0.0429
*ADD1*	rs6833874	BP response = −0.1305 −3.9535*log(Endothelin) − 5.8818*log(TRIG) + 5.0602*Gender − 7.8010*SNP	0.0566	0.0077
MODERATE				
SBP				
Gene	Polymorphism	Model^1^	PVE	*P* value
None	None	BP response = −3.2665 + 10.9273*log(INSULIN)	0.6623	–
Gene	Polymorphism	Model^1^	Partial PVE^2^	*P* value^3^
*ACE*	rs3730036	BP response = −4.1164 + 8.0972*log(INSULIN) + 11.9101*SNP	0.0690	0.0736
*CYP11B2*	rs3802228	BP response = −4.8800 + 8.2175*log(INSULIN) + 5.6512*SNP	0.0857	0.0429
*CYP11B2*	rs3802228^4^	BP response = −4.1164 + 8.0972*log(INSULIN) + 11.9101*SNPr	0.0690	0.0736
*CYP11B2*	rs6432	BP response = −4.1164 + 8.0972*log(INSULIN) + 11.9101*SNP	0.0690	0.0736

VIGOROUS, 100% of peak oxygen consumption (VO_2_peak); MODERATE, 60% VO_2_peak; SBP, systolic blood pressure; DBP, diastolic blood pressure; PVE, proportion of variance explained; *ACE*, Angiotensinogen‐Converting Enzyme, *AGTR1*, Angiotensin Type 1 Receptor, *CYP11B2*, Aldosterone Synthase, *ADD1*, Adducin; SNP, polymorphism; TRIG, triglycerides; AASICONT, resing ambulatory arterial stiffness index over 19 h; Orientation 19 h SBP, resting ambulatory SBP over 19 h.

^1^Restricted maximum likelihood estimates reported; all covariates centered except for polymorphism.

^2^Either polymorphism only (when there is no polymorphism by time interaction) or joint polymorphism and polymorphism by time effects (when there is a polymorphism by time interaction), computed using maximum likelihood.

^3^Likelihood ratio tests for either polymorphism only (when there is no polymorphism by time interaction) or joint polymorphism and polymorphism by time effects (when there is a polymorphism by time interaction) under maximum likelihood.

^4^Recessive model for polymorphism (i.e., SNPr = 0 if 0 or 1 copy of the minor allele; SNPr = 1 if 2 copies of the minor allele; additive genetic models used for all other polymorphisms (i.e., SNP = #minor allele).

### Combined annotation‐dependent depletion score

The CADD score for each variant passing the threshold for multiple testing is listed in Tables 2 (VIGOROUS) and 3 (MODERATE). *ADD1* rs16843169 had a CADD score of 13.93 and *AGTR1* rs74662294 had a CADD score of 9.038 in Table 2 and *ACE* rs3730036 had a CADD score of 17.2 in Table 3, indicating they are likely to have causal and regulatory effects (Kircher et al. 2014).

## Discussion

Because the heritability of hypertension largely resides in the kidneys, over a decade ago, we embarked upon a series of candidate gene association studies that involved renal polymorphisms *reported* to be associated with hypertension and the BP response to pharmacotherapy and/or exercise training for their associations with PEH. In these discovery phase studies, variants in *ACE*,* AGT*,* AGTR1*,* CYP11B2*, and *ADD1* exhibited exercise‐intensity associations with PEH among Caucasians (Blanchard et al. 2006; Pescatello et al. 2007, 2008; Ash et al. 2013a; Bruneau et al. 2015). Using genomic technology that was not available until recently, the major findings from this validation study were that variants in *ACE*,* AGTR1*,* CYP11B2*, and *ADD1* associated with PEH after VIGOROUS but not MODERATE among AF only. Among variants in these genes passing multiple testing thresholds, after VIGOROUS over 19 h, as the #MA increased, BP *decreased* by 12–30 mmHg after exercise compared to control. The magnitude of these PEH by renal genotype reductions is much greater than that reported for PEH on average (Pescatello et al. 2015a,b), and equals or exceeds those reported with antihypertensive drug treatment (ALLHAT Officers and Coordinators for the ALLHAT Collaborative Research Group. The Antihypertensive and Lipid‐Lowering Treatment to Prevent Heart Attack Trial 2002, Brown et al. 2013). In contrast, an unexpected finding was that among *ACE* and *CYP11B2* variants passing multiple testing thresholds, after MODERATE over 19 h, as the #MA increased, BP *increased* by 21–22 mmHg. Collectively, these renal variants explained from 3% up to 9% of the variance in the BP response to acute aerobic exercise. There were no significant renal variant associations with the BP response to VIGOROUS or MODERATE among Caucasians.

AF have the highest prevalence of hypertension compared to other ethnic groups and suffer from a disproportionate burden of the adverse cardiovascular health effects associated with hypertension (Mozaffarian et al. 2014). The BP response to antihypertensive pharmacotherapy differs among AF and Caucasians as do the pharmacogenetic association patterns for the treatment of hypertension (Turner et al. 2012; Reiter et al. 2015; Do et al. 2016). Evidence also exists that PEH differs between AF and Caucasians (Headley et al. 1998; Pescatello et al. 2003; Santa‐Clara et al. 2003; Bond et al. 2005; Brandon and Elliott‐Lloyd 2006; Jones et al. 2006, 2007; Enweze et al. 2007). Our results provide insight into possible reasons for the ethnic differences in PEH that may partially reside in the vasoconstrictive actions of the renal system as modulated by the RAS and other renal structural variants and exercise intensity. For, only when the SBP and DBP responses over 19 h after VIGOROUS and MODERATE were examined by renal variant #MA did ethnic differences appear. These findings are consistent with our previous reports of exercise intensity‐dependent associations of renal polymorphisms with PEH (Blanchard et al. 2006; Pescatello et al. 2007, 2008; Ash et al. 2013a). Yet, it is important to note that although the same genes emerged from our discovery phase and validation studies regarding their associations with the BP response to acute aerobic exercise (i.e., *ACE*,* AGTR1*,* CYP11B2*, and *ADD1*), the individual variants harbored within them differed between studies, and we did not confirm our original findings in Caucasians as we only observed significance among AF in this study.

An important finding from both our discovery phase and replication studies is that established clinical biomarkers still account for most of the PVE in PEH (Pescatello et al. 2008; Eicher et al. 2010; Ash et al. 2013a). Indeed, clinical covariates that included resting ambulatory BP, resting ambulatory arterial stiffness index, age, gender, fasting insulin and triglycerides, and endothelin accounted for 66.2–92.5% of the variation in the BP response after VIGOROUS or MODERATE depending on the final multivariable regression model. Nonetheless, individual renal variants accounted for an additional 3–9% of the PVE in the BP response after VIGOROUS or MODERATE, a partial PVE that is larger than that typically reported for individual variants in exercise genomic studies examining health‐related phenotypes (Ash et al. 2013a; Bruneau et al. 2015).

An interesting observation is that the same subject carried one or two copies of the Minor Alleles (MA) from each of the renal variants associated with the BP response after VIGOROUS (Table 2) or MODERATE (Table 3), but these two subjects were different people depending upon the intensity of bout. Indeed, one subject could be labeled a “super” responder to VIGOROUS with an average SBP reduction of 30 mmHg and DBP reduction of 20 mmHg over 19 h. While the other subject could be considered an “adverse” responder to MODERATE with an average SBP increase of 16 mmHg and DBP increase of 4 mmHg over 19 h. Collectively, our findings illustrate the complexity and challenges of using genomic information to inform clinical decision making regarding the future use of personalized exercise prescriptions to maximize the effectiveness of exercise as antihypertensive therapy.

A major limitation of this study is the small sample size so our findings should be interpreted with caution. For this reason, we calculated the power of the variant screening method using simulation based on the estimated parameters from the screening model for each significant renal variant in Tables 2 and 3, and calculated the power to detect BP‐genotype significant differences as the proportion of 1000 simulations in which the variant *P*‐value (calculated using the residual degree of freedom method) was less than alpha=0.05/300 (i.e., adjusted for 300 unique genotypic profiles for AF). The power to detect the BP‐genotype significant differences ranged from 19.5% for *CYP11B2* rs6432 in Table 3 to 82.4% for *ACE* rs1055086 in Table 2. In instances where there was insufficient power, for example *ADD1* rs16843169 with a power of 26.5% and CADD score of 13.93 in Table 2, the CADD score nonetheless often indicated that particular variant was likely to have causal and regulatory effects.

In addition, we instituted methodological strategies to increase the statistical power to detect renal genotype‐BP associations should they exist (Bouchard 2011; Ash et al. 2013a; Bruneau et al. 2015; Mattsson et al. 2016). These strategies included a repeated measure design among the same individuals that increased the number of observations per subject by 19 hourly time points, a focused inquiry of polymorphisms with a prioritized panel of genes that reduced the search space within the genome, performing high throughput exon sequencing to focus on functional regions of the gene, and inclusion of the same standardized protocols and methods in our discovery phase and replication studies. Other strengths of this replication study were inclusion of a randomized control design with the subjects serving as their own control; the gold standard of BP assessment, ambulatory BP monitoring (Niiranen et al. 2014); and a well‐controlled and monitored exercise exposure; as well as adjustment for multiple testing that was based only on genetic variants exhibiting variability in the #MA and with unique genotypic values. Yet, due to the small sample size and in instances when only one or two subjects carried a MA, the multivariable models adjusted for covariates may have been over fit to accommodate those subjects, our findings should be regarded as preliminary and interpreted with caution.

In conclusion, in a replication cohort using high‐throughput exon sequencing, we found that renal genes from our prior work once again exhibited exercise intensity‐dependent associations with the ambulatory BP response to acute bouts of aerobic exercise. Our findings are preliminary and are limited by a small sample size, therefore, they should be interpreted with caution. Nonetheless, they are exciting because they add new and novel information to the exercise genomic literature regarding the immediate antihypertensive effects of exercise. Future work should utilize a multi‐level “omics” approach involving a focused inquiry of genes related to renal function and other BP regulatory functions and their transcript and proteomic targets among a large, ethnically diverse sample of adults with hypertension to better inform clinical decision making regarding the nuances of the prescription of exercise as antihypertensive lifestyle therapy.

## Conflict of Interest

None declared.

## Supporting information




**Appendix S1.** The prioritized panel of genes and their associated polymorphisms that were sequenced.
