# Supplementary material for: Deep‐targeted exon sequencing reveals renal polymorphisms associate with postexercise hypotension among African Americans
Source: Physiol Rep. 2016 Oct 10;4(19):e12992. doi: 10.14814/phy2.12992 (PMC5064144; doi:10.14814/phy2.12992)

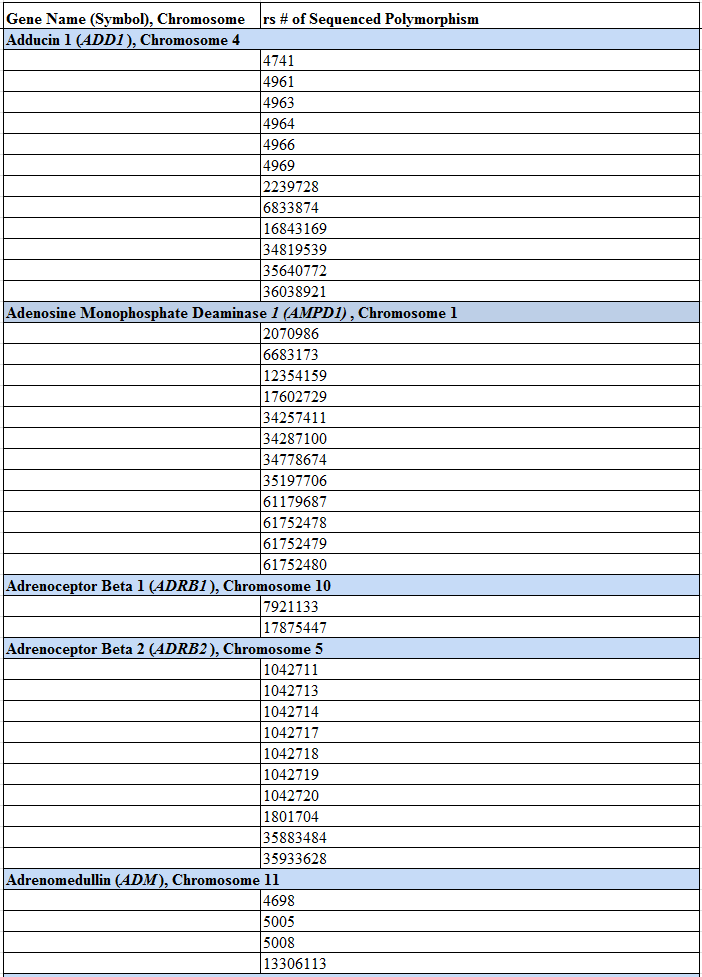


Supplemental Material Appendix. The Prioritized Panel of Genes and Their Associated Polymorphisms that Were Sequenced


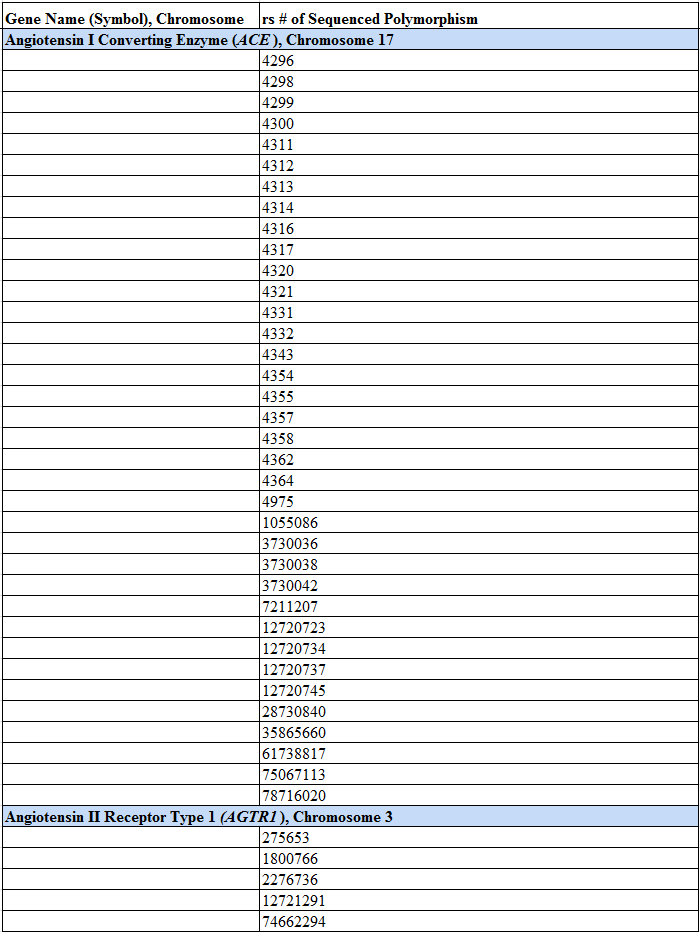


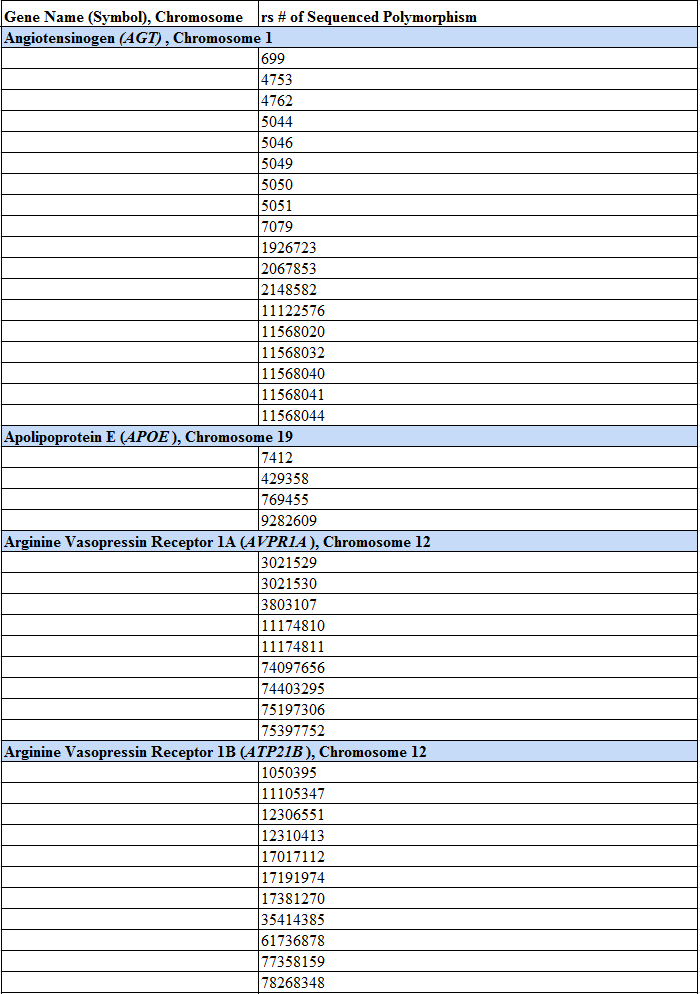


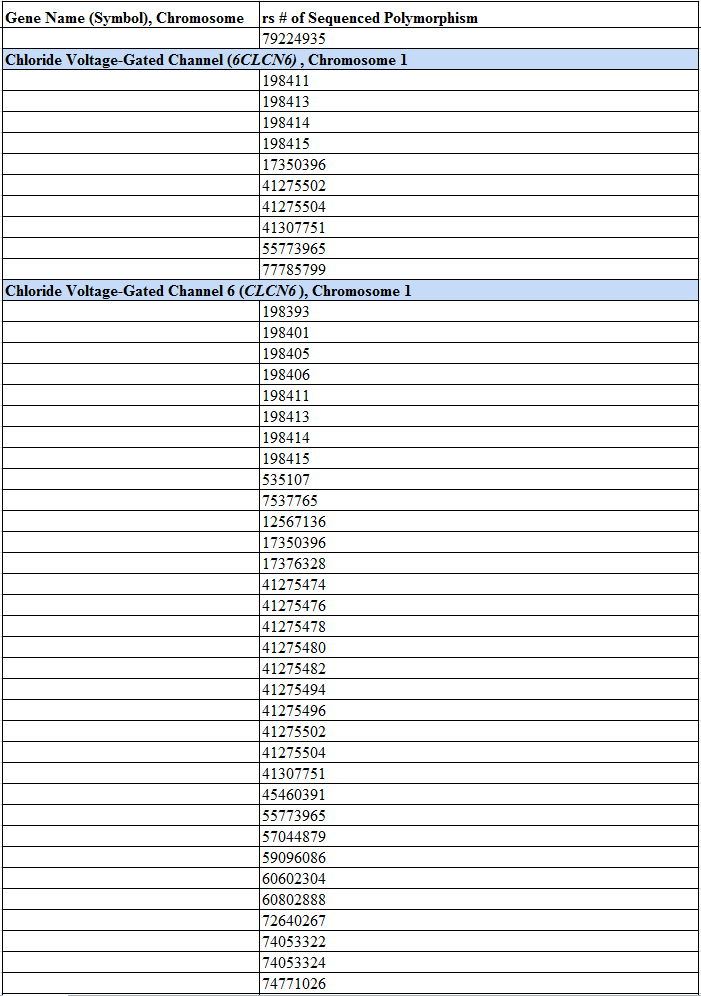


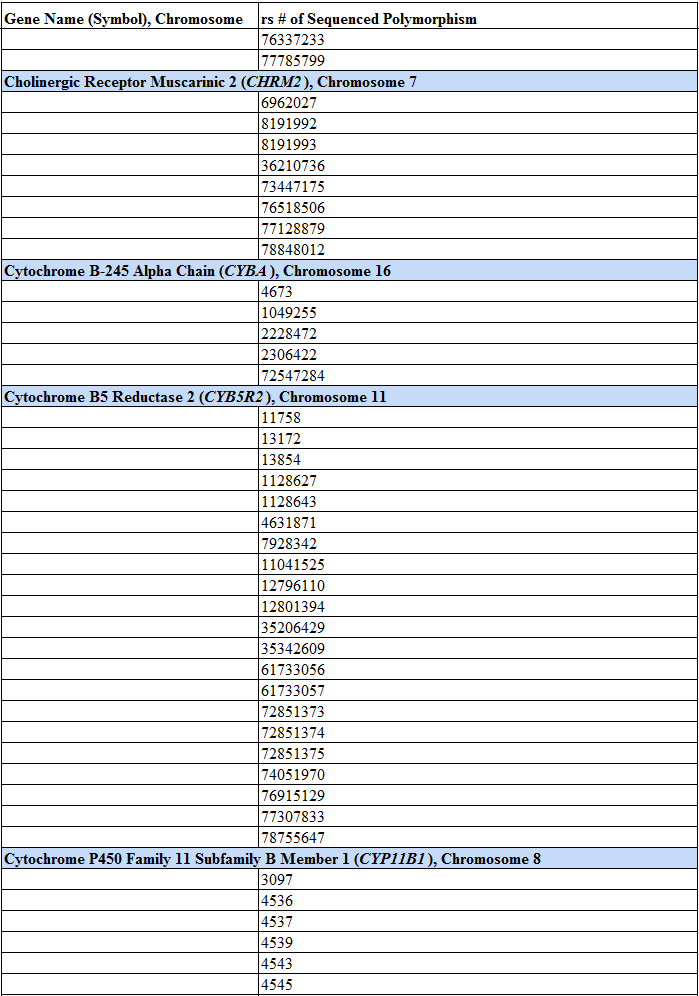


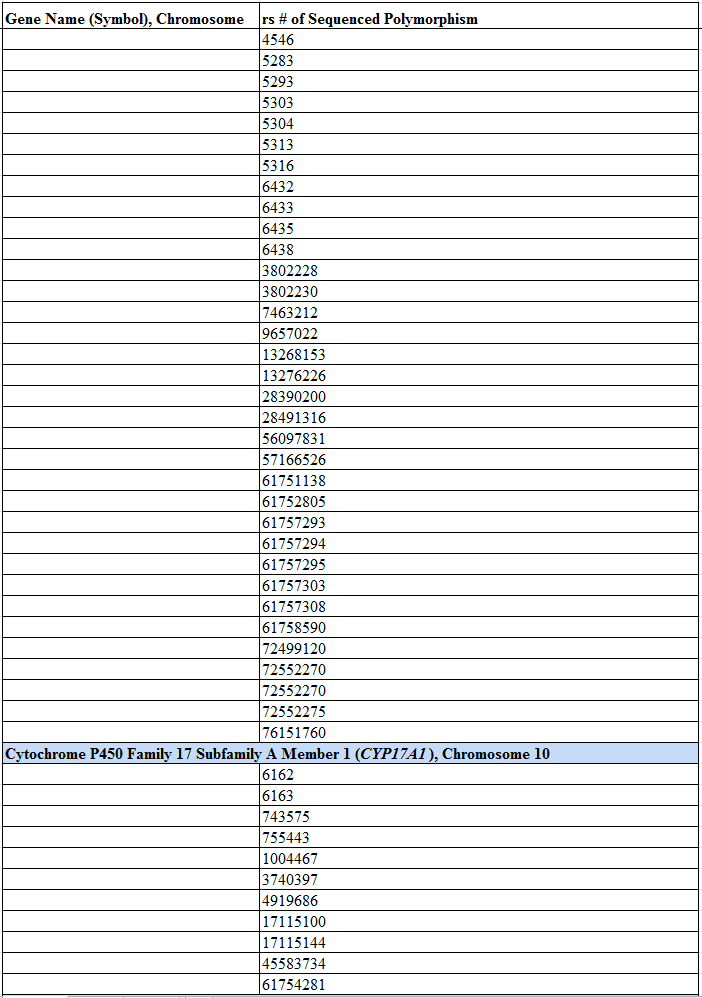


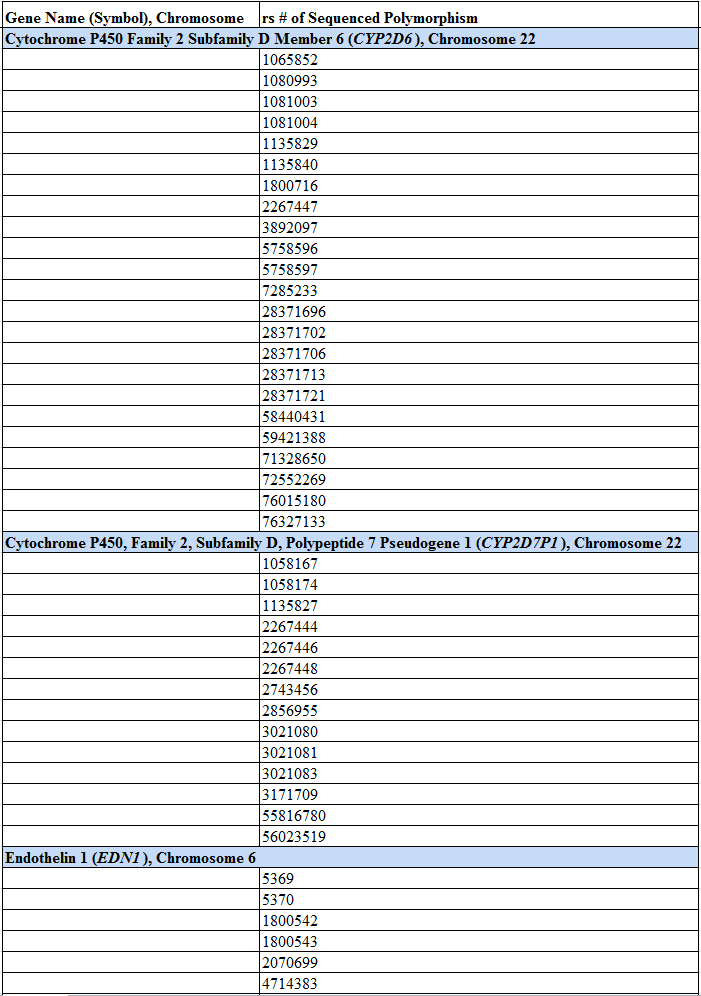


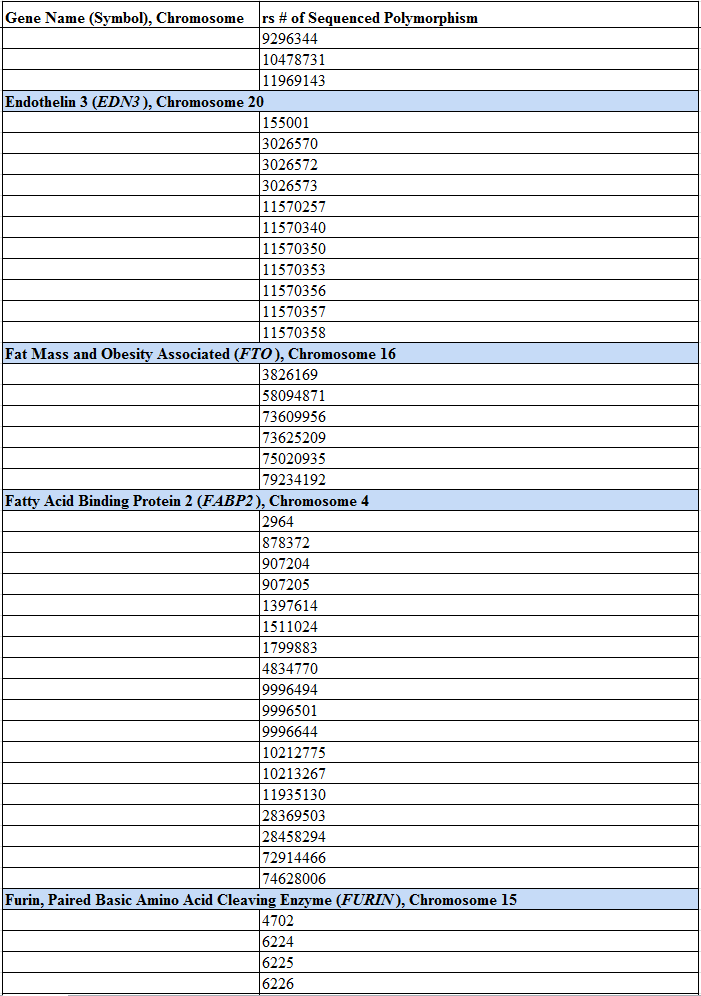


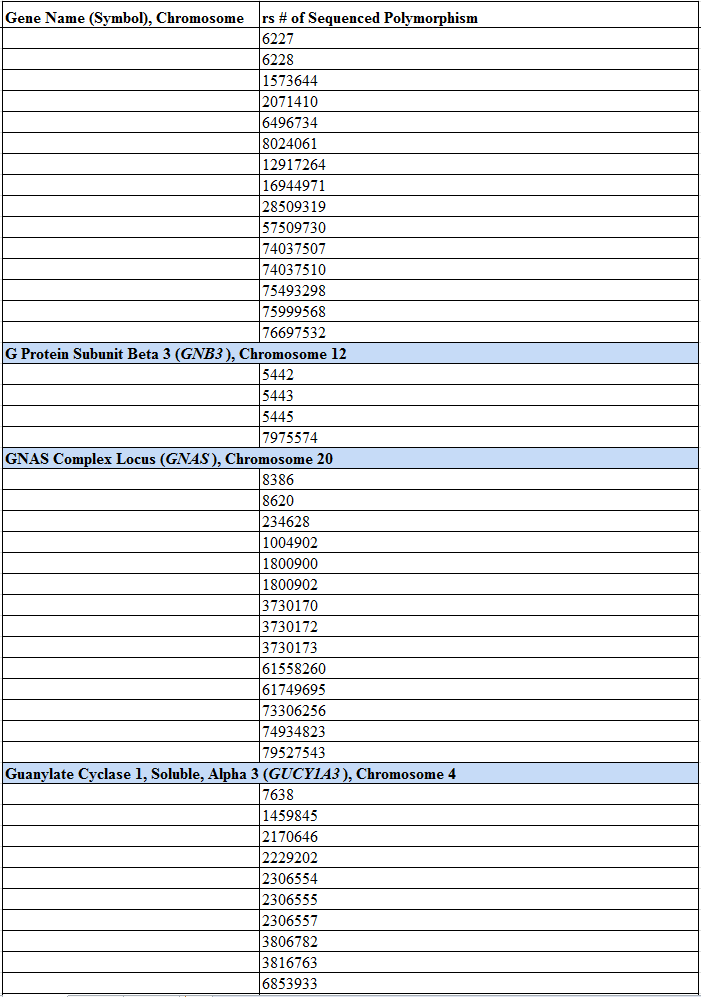


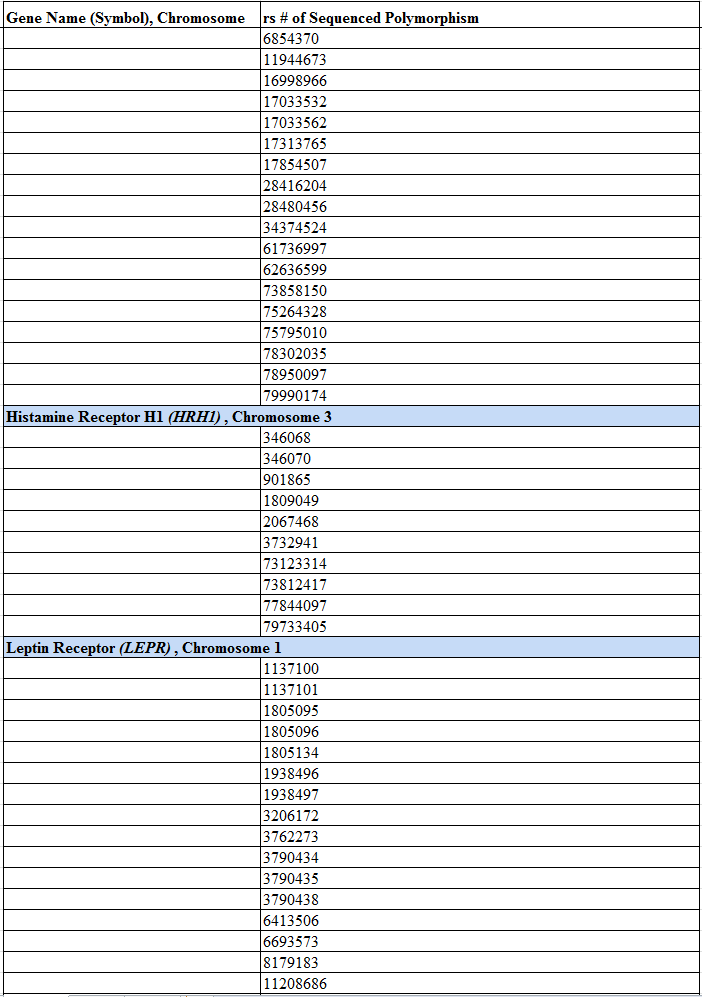


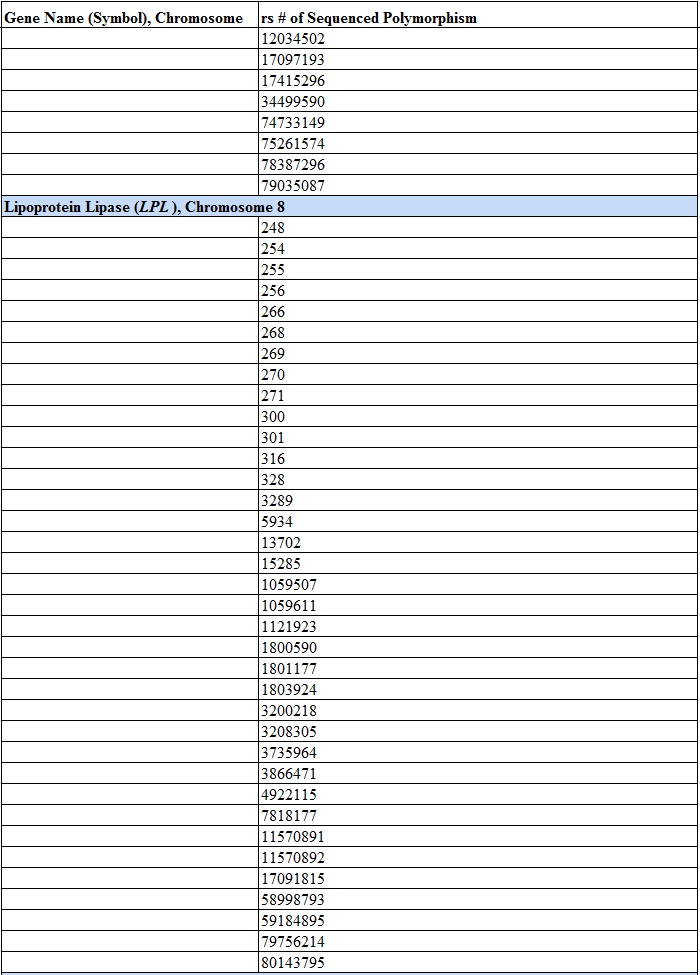


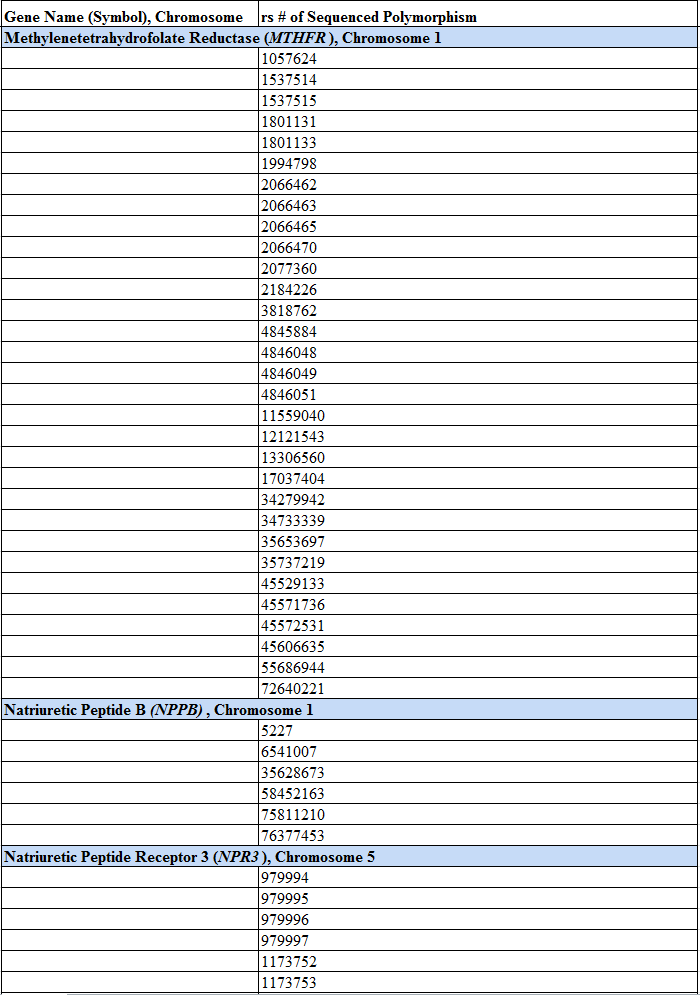


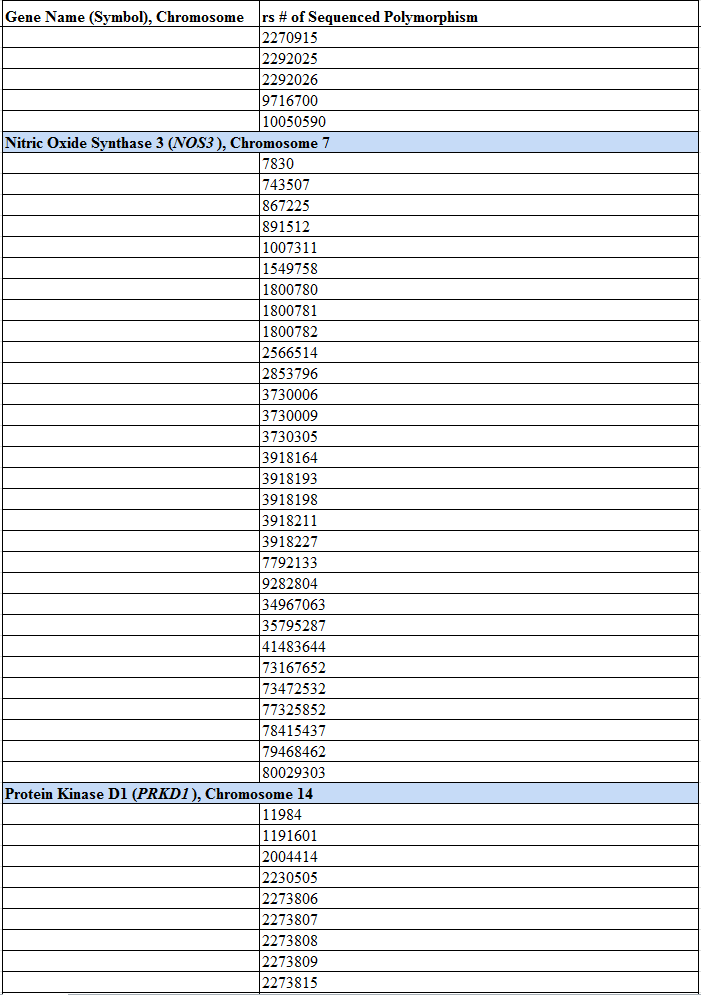


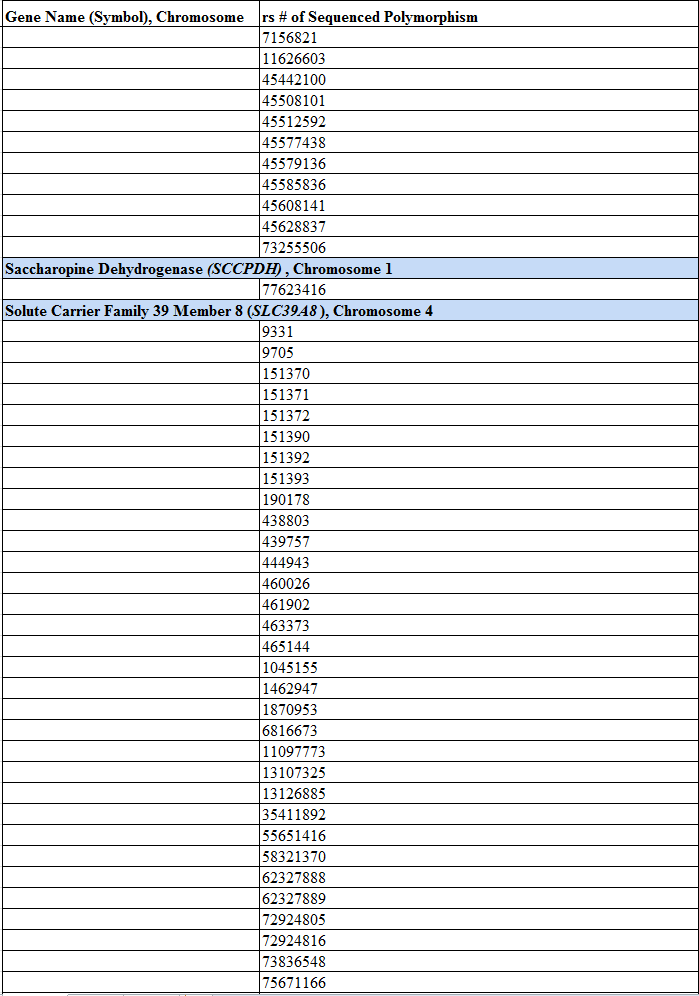


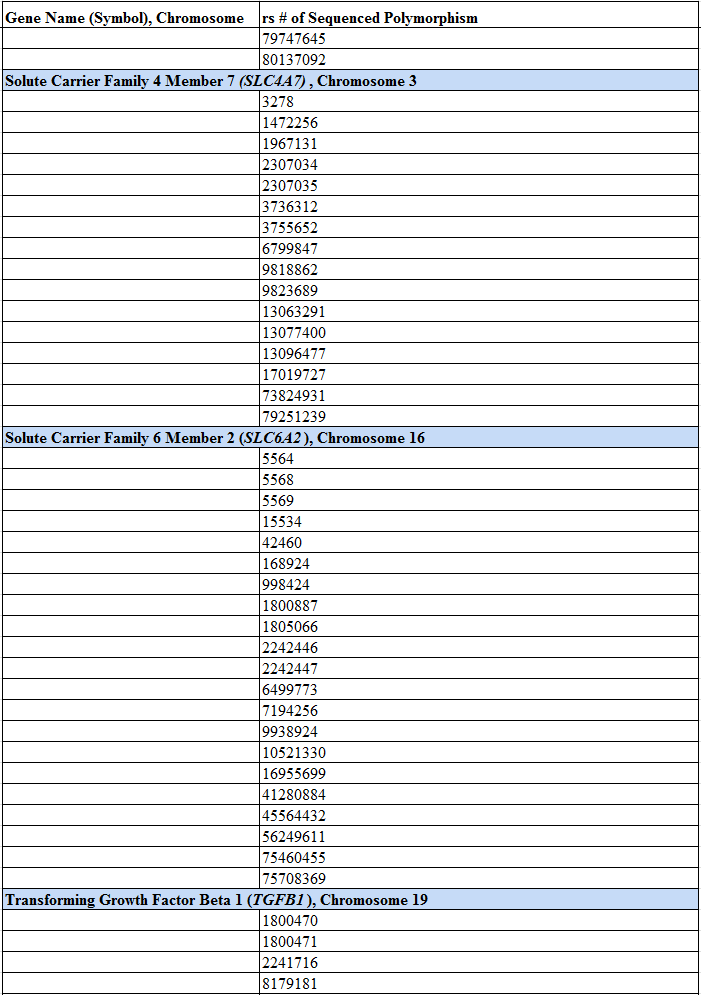


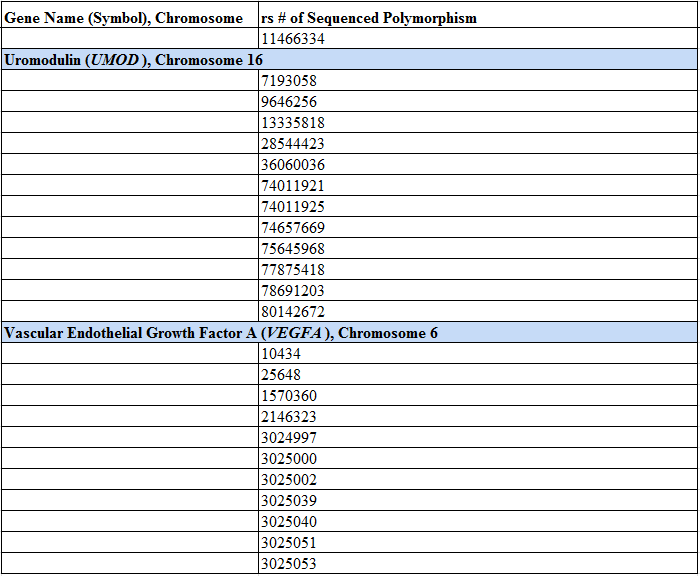

Supplement: Supplementary file 1 — Appendix S1. The prioritized panel of genes and their associated polymorphisms that were sequenced. [file PHY2-4-e12992-s001.docx]
